# Cervical dystonia: effectiveness of a standardized physical therapy program; study design and protocol of a single blind randomized controlled trial

**DOI:** 10.1186/1471-2377-13-85

**Published:** 2013-07-15

**Authors:** Joost van den Dool, Bart Visser, J Hans TM Koelman, Raoul HH Engelbert, Marina AJ Tijssen

**Affiliations:** 1 Department of Neurology, University Medical Centre Groningen, University of Groningen, Hanzeplein 1, Groningen, 9700 RB, The Netherlands; 2 Department Exercise Therapy, Amsterdam School of Health Professions, Tafelbergweg, Amsterdam, 51,1105 BD, The Netherlands; 3 Department of Neurology, Academic Medical Centre Amsterdam, Meibergdreef 9, Amsterdam, 1100 DD, The Netherlands; 4 Department of Physiotherapy, Amsterdam School of Health Professions, Tafelbergweg 51, Amsterdam, 1105 BD, The Netherlands; 5 Department of Rehabilitation, Academic Medical Center, Meibergdreef 9, Amsterdam, 1100 DD, The Netherlands

**Keywords:** Cervical dystonia, Spasmodic torticollis, Physical therapy, Botulinum toxin, Activities of daily living, Quality of life

## Abstract

**Background:**

Cervical dystonia is characterized by involuntary muscle contractions of the neck and abnormal head positions that affect daily life activities and social life of patients. Patients are usually treated with botulinum toxin injections into affected neck muscles to relief pain and improve control of head postures. In addition, many patients are referred for physical therapy to improve their ability to perform activities of daily living. A recent review on allied health interventions in cervical dystonia showed a lack of randomized controlled intervention studies regarding the effectiveness of physical therapy interventions.

**Methods/design:**

The (cost-) effectiveness of a standardized physical therapy program compared to regular physical therapy, both as add-on treatment to botulinum toxin injections will be determined in a multi-centre, single blinded randomized controlled trial with 100 cervical dystonia patients. Primary outcomes are disability in daily functioning assessed with the disability subscale of the Toronto Western Spasmodic Torticollis Rating Scale. Secondary outcomes are pain, severity of dystonia, active range of motion of the head, quality of life, anxiety and depression. Data will be collected at baseline, after six months and one year by an independent blind assessor just prior to botulinum toxin injections. For the cost effectiveness, an additional economic evaluation will be performed with the costs per quality adjusted life-year as primary outcome parameter.

**Discussion:**

Our study will provide new evidence regarding the (cost-) effectiveness of a standardized, tailored physical therapy program for patients with cervical dystonia. It is widely felt that allied health interventions, including physical therapy, may offer a valuable supplement to the current therapeutic options. A positive outcome will lead to a greater use of the standardized physical therapy program. For the Dutch situation a positive outcome implies that the standardized physical therapy program forms the basis for a national treatment guideline for cervical dystonia.

**Trial registration:**

Number Dutch Trial registration (Nederlands Trial Register): NTR3437

## Background

**Figure 1:**
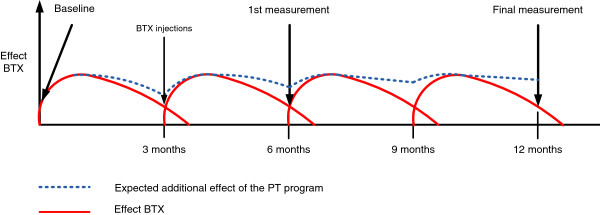
**Effect of BTX and expected additional effect of PT.** Effect of BTX. Increasing lines indicate a better effect of BTX and less severity of CD, pain and disability to perform daily life tasks. Red lines illustrate the normal effect of BTX, blue dotted lines illustrate the expected additional effect of the PT program.

Cervical Dystonia (CD), or torticollis, is a disabling neurological disorder characterized by abnormal positions of the head due to involuntary muscle contractions of the neck [1]. The posture in CD patients can feature one or a combination of postures: rotation (torticollis); lateral tilting (laterocollis); flexion (anterocollis); extension (retrocollis); and lateral shift. With an estimated prevalence of 5.7 patients per 100.000 persons in Western Europe, CD is the most common form of primary adult onset dystonia which usually starts after the age of 30 [2]. Pain is experienced in two-thirds to three-quarters of patients and is a major source of disability, which is strongly associated with the presence of muscle contractions and head deviations [3-6]. Decreased self-efficacy, fatigue, anxiety and depression are other factors associated with disability in cervical dystonia [7]. Research on focal dystonia’s, including CD, revealed abnormalities in basal ganglia function, cerebellar function, sensory processing, motor inhibition, neuro-plasticity and somatotopic cortical organisation but the pathophysiology remains largely unclear [8]. Treatment options for CD are mainly symptomatic, aiming to reduce involuntary movements, correct abnormal head positions and reduce pain. Currently, the best evidence based treatment option is to inject the dystonic neck muscles with botulinum toxin (BTX) [9-12]. The effects of BTX fluctuate over time. A peak effect occurs within 2–4 weeks after injections and is followed by a decrease of effect and return of symptoms. On average new injections are given within 12–14 weeks after the previous injections (Figure 1) [13].

In addition to BTX treatment, many CD patients in the Netherlands are referred for physical therapy (PT). However, due to the rarity of CD, experience among Dutch PT’s is lacking. Besides, the evidence for the effects of PT on the ability to perform activities of daily living in CD is very limited. [14] Only two small Randomized Controlled Trials (RCT) and one open controlled study investigated the effects of a PT program on CD [15-17]. All studies compared BTX treatment in combination with a PT program versus BTX treatment alone. All studies showed significant better scores on pain and disability in the groups receiving BTX treatment with an additional PT program. The PT programs in all three studies consisted of intense motor learning exercises (postural control, balance, strengthening axial musculature and facilitation of voluntary movement), and mobilization techniques of the cervical spine and dystonic muscles. PT programs varied from 40 minutes per session every other day for six weeks [16], 75 minutes per session 5 days a week for five weeks [17] up to 90 minutes a day for 2 weeks [15]. Although the results of these PT treatments were positive, it is difficult to implement them to current regular care of chronic diseases provided by physiotherapists and exercise therapists. For most patients and therapists it will not be feasible to combine such an intensive program with their daily lives and practice.

One approach towards the treatment of CD was suggested by the French physiotherapist J.P. Bleton [18]. The main goals of this program are the rehabilitation of the antagonist muscles and the control of the dystonic movements by frequent training in a functional context. Exercises are taught during one or two PT sessions a week. After teaching the patients, intensive training is required in the patient’s environment (up to 10 times a day for 10 minutes). In addition, patients are encouraged to correct the dystonic posture during their daily live activities by turning their head in the opposite direction of the dystonic posture. Eventually, patients should be able to control the dystonic movements independently. This approach with increasing control of the patients and decreasing therapist involvement seems more applicable than the intensive programs by Tassoreli, El Bahwrady and Queiroz [15-17]. The effect of the Bleton treatment has never been investigated in a large randomized controlled trial. Not only for practical reasons, but also based on the pathophysiological knowledge of CD, a longer treatment period with a more functional approach and independent continuation of the treatment program seems more appropriate for the long term benefits of PT [15].

In the current study we developed a PT program with elements of the approach suggested by Bleton. We added current knowledge of motor (re) learning principles, coaching and principles of providing feedback in a standardized PT treatment program (Table 1). This resulted in a standardized, tailored PT program comprising of a one year training that aims to improve the ability to perform daily life tasks by emphasizing independent training in the patient’s own environment [18-20]. The standardized PT treatment program was developed according the AGREE standards [21] in corporation with the Royal Dutch Society for Physiotherapy (KNGF), the Society for Cesar- and Mensendieck Exercise Therapy (VvOCM) and the Amsterdam School of Health Professions (ASHP). The standardized PT program was developed within the DystonieNet, a national collaboration between neurologists and allied health professionals in research, education and treatment of CD, which was initiated by the neurology departments of four university hospitals in the Netherlands [22]. The standardized PT program aims to relearn or adopt alternative/new movement strategies to improve activities in daily life situations.

**Table 1:** Theoretical background of the standardized PT program


**Muscle stretching /relaxation and mobilisations (de Morree**[43]**, Fung**[44]**)**		
*Principle*	*Explanation*	*Application in standardized PT program*
1. Passive mobilisation of the neck	Passive mobilization techniques of the neck create stress relaxation in the collagen fibers of the cervical facet joints. This helps to increase ROM	Passive mobilisation techniques are applied by PT’s
2. Muscle stretching for relaxation	Stretching elongates the dystonic muscle and helps to relax it due to the Golgi tendon reflex.	Passive stretching of dystonic muscles
**Motor learning principles (Kleim** &**Jones**[20]**)**		
*Principle*	*Explanation*	*Application in standardized PT program*
1. Use it or lose it	Failure to drive specific brain functions can lead to functional degradation.	Activation of antagonists
2. Use it and improve it	Training that drives a specific brain function can lead to an enhancement of that function.	Training of antagonists in order to improve voluntary movement of the head
3. Specificity	The nature of the training experience dictates the nature of the plasticity.	Functional training of activities of daily living tailored to the patients needs
4. Repetition matters	Induction of plasticity requires sufficient repetition.	Exercise of newly gained tasks 5–10 times a day for 10–15 minutes
5. Intensity matters	Induction of plasticity requires sufficient training intensity.	Training intensity is tailored for the individual and monitored over time
6. Time matters	Different forms of plasticity occur at different times during training.	1 year of therapy is divided in 3 stages according the 3 stages model of Fitts & Postner [45]
7. Salience matters	The training experience must be sufficiently salient to induce plasticity.	Functional training of activities of daily living tailored to the individual needs of the patient
8. Age matters	Training-induced plasticity occurs more readily in younger brains.	
9. Transference	Plasticity in response to one training experience can enhance the acquisition of similar behaviors.	Functional training of activities of daily living tailored to the patients needs and variation and random practice
10. Interference	Plasticity in response to one experience can interfere with the acquisition of other behaviors.	
**Transference and generalization (Shea** &**Morgan**[45]**, Schmidt** &**Lee**[46]**)**		
*Principle*	*Explanation*	*Application in standardized PT program*
1. Random practice	Enhances the transference and generalization of a task	Tasks or exercises are given in a random order
2. Variation of practice	Enhances the transference and generalization of a task	Specific tasks or exercises are performed in different contexts
**Feedback (Shea et al.**[47]**, Schmidt** &**Lee**[46]**)**		
*Principle*	*Explanation*	*Application in standardized PT program*
1. Summary Knowledge of Results	Feedback is essential for learning to take place. Summary KR is that KR is given after an entire set of trials during an exercise instead of after each individual trial. It is the most effective form for the retention and transference of a task.	Feedback is given after each set of trials of a task. Each task is performed at least 5 times after feedback is provided
**Self management (Fitts** &**Posner three-stage model**[48]**)**		
*Principle*	*Explanation*	*Application in standardized PT program*
1. Cognitive phase	The learner is concerned with understanding a task and developing strategies to perform a task and how the task can be evaluated. These efforts require a high degree of cognitive activity	During the first month patients receive 2 PT sessions a week to (re)learn and understand movement strategies. Movement strategies will be practiced at home 5–10 times a day for 10–15 minutes
2. Associative phase	The learner has selected the best strategy for a task and starts to refine it. This stage requires less cognitive activity	During this stage patients receive 1 PT session. Movement strategies from the first stage will be increased in difficulty. Movement strategies will be practiced at home 5–10 times day for 10–15 minutes
3. Autonomous phase	The learner is able to perform a skill automatically. A low degree of attention is required.	During the last (autonomous) stage, patients are encouraged to perform the learned tasks independently and to improve and maintain their (re)gained abilities themselves. Therapists will have a coaching role. Patients receive one PT session a month for additional advice and motivation.

The primary objective of this study is to evaluate the effectiveness of the standardized PT program on improving the ability to perform daily life activities in CD patients compared to usual PT that is given in Dutch private practices. Both PT programs are add-on treatment to BTX-injections. Measurement will take place just prior to the BTX injections as it is hypothesized that the effects of the PT program will mainly occur in the period between the injections when the BTX wears off and symptoms return (Figure 1). Secondary objectives are to evaluate the effects on severity of CD pain, quality of life, anxiety and depression.

In addition, cost effectiveness will be detremined by comparing the costs and health utility of the new standardized PT program with the care as usual PT treatment. It is hypothesized that the standardized PT program will be more cost effective and more effective in improving the ability to perform daily life tasks of CD patients than regular PT.

A positive outcome of this study will lead to the development of a national treatment guideline which will be implemented via the Dutch DystonieNet.

## Methods/design

### Study design

The study will be conducted as a multi-center single blind randomized controlled trial in three Dutch university hospitals. Patients will be randomly assigned to the experimental group or control group using a computerized randomization protocol. Patients in the expe-rimental group will be referred to specialized PT’s who are trained prior to the study to perform the standardized PT program. Patients in the control group will be referred to regular PT’s and receive care as usual. All data will be collected at baseline, after six months and after one year. In order to determine the additional effects of a PT program, measurements will be performed briefly before the BTX injections at the outpatient clinics of the hospitals. This implies that we measure the effect of the PT program in a period that BTX has the least effect on the symptoms of dystonia (Figure 1). Measurements will be performed by a blind and independent assessor since it is impossible to blind the therapists and patients for treatment allocation.

### Participants

The study aims to include 100 patients with primary CD of 30 years and older, stable on BTX treatment for more than one year. Exclusion criteria are secondary or hereditary forms of dystonia, dystonia in other bodyparts than the neck and patients who had surgery for the treatment of dystonia.

### Interventions

#### Standardized, tailored PT program

Subjects in this group receive a one year PT program according the standardized PT program in combination with BTX injections. The PT program will start two weeks after the injections. The emphasis of the PT program is on the functional performance of the exercises adapted to daily life situations, muscle stretching, passive mobilization of the neck and training principles which have found to be relevant for neural rehabilitation and motor learning and will be performed by trained physical therapists [18-20]. A summary of the theoretical basis with respect to *‘muscle stretching and mobilizations’ , ‘motor (re)learning’, ‘transference and generalization’, ‘feedback’ and ‘self management’,* is displayed in Table 1.

#### Regular PT

Subjects randomized in this group will receive BTX injections and regular PT once a week for a period of one year. In contrast to the standardized PT program, interventions are not given by specialized therapists. Due to the rarity of CD, the average therapist in the Netherlands has little knowledge about CD. It is likely that common interventions like massage, stretching and exercise of the dystonic muscles are used. Specific information of the weekly sessions and treatment will be retrieved from the local PT’s after the patients finished the study period of one year.

### Outcome variables

#### Disability

Disability as measured with the disability subscale of the Toronto Western Spasmodic Torticollis Rating Scale (TWSTRS) is the primary outcome of this study. The TWSTRS scale is a widely used scale in research and is a valid and reliable tool to measure severity, disability and pain in CD (Kendall Tau = 0.85, p < 0.01) [23,24]. The disability section is a six point Likert scale which consists of six items like driving a car, reading and performing ADL activities (max 30 points). Lower scores indicate less disability.

Disability will also be measured with the Functional Disability Questionnaire (FDQ). The FDQ is a 27 item scale to measures the impact of CD on daily functioning. Questions are asked about the extent to which CD affects the engagement in and performance of a sample of activities at the present time. Each item is rated on a 5-point scale (maximal score 68 points) The FDQ has a high reliability (r=.93, P<0.001) [25].

#### Severity of CD

Severity of CD will be measured with the Tsui scale [26]. The Tsui scale measures different aspects of abnormal posture and movements in CD patients. It has a maximal score of 25 points. The Tsui-scale is a widely used, standardized and reliable scale (ICC=.86) to measure the severity of CD [26] Lower scores indicate less severity of dystonia.

Severity of CD will also be measured with the Clinical Global Impressions-Severity of Illness Scale (CGI-S) and the Clinical Global Impression - Improvement scale (CGI-I). Both scales are observer- or patient- rated scale that measure illness severity and global improvement on a 7-point scale. Both scales are widely used and reliable and validated for a numerous of disorders (r=0.41 to 0.77, p=0,05 for self perceived measures and r=0.36 to 0.84, p=0.05 of clinician administered measures of anxiety, depression, impairment and quality of life) [27,28].

#### Active range of motion

To determine the changes in the ability to perform voluntary movements, active range of motion (AROM) will be measured with a cervical range of motion meter (CROM) [29]. The CROM is a frame that will be placed on the head with three separate inclinometers to measure AROM in the sagittal, coronal and horizontal planes. First the resting position of the head will be measured and subsequently the AROM of flexion, extension, lateral flexion and rotation. Although the psychometric properties of the CROM in patients with CD are unknown, in a healthy population the CROM is a reliable instrument to measure cervical ROM (intratester reliability ranged .63-.93 intertester reliability ranged .74 -.87) [29]. To determine the additional effects of PT on pain, patient are asked to rate their pain on a Numeric Rating Scale (NRS). A score of 0 means no pain and a score of 10 means the worst pain imaginable. The NRS is a validated and reliable tool for the assessment of pain (Spearman r =.94 between VAS and NRS, test-retest reliability ICC = .90) [30,31].

#### Quality of life

Quality of Life (QoL) will be measured with the Cranio-cervical Dystonia Questionnaire (CDQ-24) and Short Form 36 (SF-36) [32,33]. The CDQ-24 is a validated and disease specific, self reporting questionnaire to evaluate quality of life of patients with cervical dystonia on a five point likert scale [32]. The CDQ-24 subscales showed moderate to high correlations with those SF-36 subscales measuring similar aspects (Pearson’s correlation r = 0.50–0.73; P<0.001, each).The score ranges from 0 to 96 points where lower scores indicate a better QoL. The SF-36 is a validated generic measure for QoL containing 36 items measuring eight dimensions of health [33]. Scores of the different dimensions can range from 0 (worst) to 100 (best).

#### Anxiety and depression

Since 25 to 59% of the CD patients suffer from anxiety disorders or depression [25,34], the effects of PT are determined with the Beck’s Anxiety Index [35] and Beck’s Depression Index [36,37]. Both instruments are validated and reliable tools and are rated on a 21 item 4 point Likert scale (BDI: r = .73 with Hamilton Psychiatric Rating Scale for Depression. BAI: test-retest reliability coefficient of .67, R=.54, p=0.05 with anxiety).

#### Cost effectiveness

To determine the cost effectiveness of both physical therapy programs, the costs per quality adjusted life year (QALY) will be calculated. In addition, cost-effectiveness related to the clinical outcome will be calculated, with the costs per unit on the TWSTRS-disability scale as the outcome measure. Costs which are associated with loss of productivity due to disability or inability to work will be registered in the subgroup of patients below the age of 65 with the Productivity Costs Questionnaire (PCQ) and EuroQoL-5D (EQ-5D). The PCQ is a 22 item generic questionnaire used to measure absence of work due to health problems and is advised as standard instrument for use in economic evaluations of Dutch healthcare [38,39]. The EQ-5D is a six item, standardized measure of health status in order to provide a simple, generic measure of health for clinical and economic appraisal [39,40].

### Sample size

The power calculation is based on the study by Brans et al. investigating the long term effect of BTX on disability and functional health [41]. This study showed an average improvement of 7.1 out of 30 points on the disability subscale of the TWSTRS after 1 year of BTX treatment in CD patients. It is estimated that the additional effect of the PT program according the treatment guideline will be at least half the effect caused by BTX. The cut off for the success of the PT program is therefore, set on an average improvement of 3.5 out of 30 points on the TWSTRS disability scale which is clinically relevant according Brans et al. [42]. With a power of 0.80 and an alpha of 0.05, each group will need 44 subjects. With a loss of 10% taken into account, 50 subjects in each group are required.

### Analysis

Differences in all outcome measures, with exception of the measures for cost effectiveness, will be determined with a mixed between-within (repeated measures) analysis of variance for both treatment arms, across three time periods (baseline, after six months and one year). All analyses will be performed under the intention to treat principle in SPSS 20.0. Differences will be considered significant at p-value < 0.05.

The cost effectiveness will be determined by a cost-utility analysis from a societal perspective with a time horizon of one year. Cost-utility analysis facilitates the comparative assessment of health care innovations across different types of interventions, disease areas and health care settings. Incremental cost-utility and cost-effectiveness ratios for the add-on standardized PT program versus add-on regular PT will be calculated as the extra costs per QALY gained and the extra costs per unit decrease in TWSTRS-disability score. The cost effectiveness will be calculated according the most recent guidelines for unit costing in healthcare research [40]. The friction cost method will be applied to calculate the costs of production loss as measured with the PCQ, EQ-5D. Unit costs of production loss will be based on the most recent national guidelines for unit costing in healthcare research [40]. The base year for unit costing will be 2013.

### Ethical considerations

In accordance with the local medical ethics committee (MEC) guidelines, written informed consent is required from participants who fulfil the selection criteria. The study has been approved by the Medical Ethics Committee of the Academic Medical Center, Amsterdam (MEC 2012_048). This study is registered under Trial registration number NTR3437 of the Dutch trial registration (Nederlands Trial Register).

## Discussion

In our study we aim to fill the gap in evidence based medicine to treat CD patients with PT by performing a large RCT towards the (cost) effectiveness of a standardized, tailored PT program. There are several differences of this study compared to studies reported in the current available literature.

Although other studies have showed added value of short, high intensity PT program on pain and disability in combination with BTX treatment, follow-up measurements were not performed and therefore it is not known if a wash out of treatment effects will occur [15-17]. Since CD is a life lasting disorder, a longer treatment period seems more appropriate to establish lasting changes. We therefore choose for a treatment period of one year in contrary to the other studies which lasted five weeks maximal [15-17]. Another difference with other studies is that the standardized PT program tends to teach patients themselves, how to improve their ability to perform daily life tasks and to manage their symptoms in their own environment. To establish lasting changes and the ability of patients to manage their symptoms in their own environment, we have chosen for a treatment period of one year. The standardized treatment program itself is based on modern principles about motor learning, transference and generalization of learned tasks to enhance lasting (neuroplastic) changes (Table 1) [20,43-48]. Based on these principles, we aimed for a tailored, evidence based intervention that is thought to be more effective than regular interventions.

It is hypothesized that the overall added effect of the standardized PT program on the BTX treatment lies between the periods that the BTX is starting to wear off and the BTX is starting to work again after new injections (Figure 1). Other studies performed measurements in the periods when the peak effect of BTX occurred (2–4 weeks after injections) which make it impossible to determine the additional effects of a PT program on CD [15-17]. We therefore choose to measure the effects of PT just prior to the BTX injections when the interference of BTX effects are minimal.

Another goal of the standardized treatment program is to make patients less dependent of healthcare providers and to decrease the healthcare costs for this patient group.

In the Netherlands many CD patients are referred for physical therapy. Since CD is a chronic indication for PT, patients receive (except for the first 20 treatments) unlimited reimbursement for PT which results in long lasting use of healthcare in the current, regular situation. We therefore added an economic evaluation to compare the cost effectiveness of the standardized PT program with physical therapy care as usual.

### Future implications

In the case of a positive outcome of this study, the standardized PT program will be used as a basis for a national treatment guideline which will be implemented via the Dutch DystonieNet.

## Abbreviations

CD: Cervical Dystonia; BTX: Botulinum toxin; PT: Physical therapy; RCT: Randomised Controlled Trial; KNGF: Royal Dutch Society for Physiotherapy; VvOCM: Society for Cesar and Mensendieck therapy; ASHP: Amsterdam School for Health Professions; ROM: Range of Motion; KR: Knowledge of Results; FDQ: Functional Disability Questionnaire; CGI-S: Clinical Global Impression Severity scale; CGS-I: Clinical Global Impression Improvement scale; AROM: Active range of motion; CROM: Cervical range of motion; QoL: Quality of life; BDI: Beck depression index; BAI: Beck anxiety index; SF-36: Short form 36; PCQ: Productivity cost questionnaire; EQ-5D: EuroQoL 5D.

## Competing interests

JHTM Koelman and MAJ Tijssen received an unrestricted research grant from Ipsen Pharmaceutical and Allergan Inc. for studies and teaching workshops on dystonia and from Ipsen to finance a specialized dystonia nurse. Ipsen and Allergan have no role in study design, collection, analysis, interpretation of data, in the writing of the report and in the decision to submit the paper for publication. The other authors declare that they have no competing interests.

## Authors’ contributions

JD wrote the first draft of the manuscript and BV, RE, HK and MKT contributed to the completion of the manuscript. All have made substantial contributions to conception and design of the study. All authors read and approved the final manuscript.

## Pre-publication history

The pre-publication history for this paper can be accessed here:

http://www.biomedcentral.com/1471-2377/13/85/prepub
